# Supplementary material for: Perinatal mortality after Chornobyl in contaminated regions of Ukraine
Source: PLoS One. 2024 May 20;19(5):e0303427. doi: 10.1371/journal.pone.0303427 (PMC11104673; doi:10.1371/journal.pone.0303427)
Supplement: S1 File — (DOCX) [file pone.0303427.s001.docx]

**Supporting Material**

**
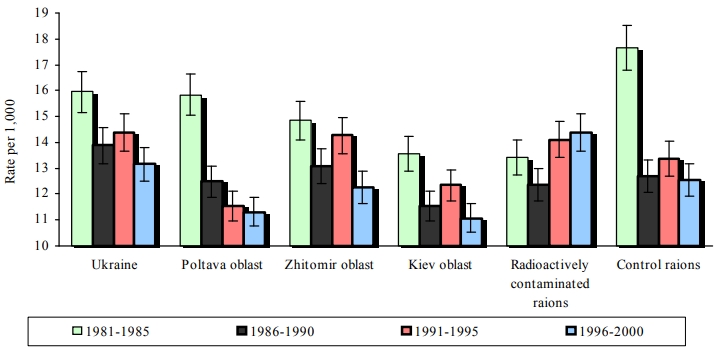
****S1 Fig. Infant mortality rates (95% CI) in Ukraine in selected periods after Chornobyl**

From the 2006 WHO report [2], page 88.

**S2 Fig. Age dependency of strontium concentration in residents of Muslyumovo in 1980.**(from Tolstykh et al. 1997 [5]).


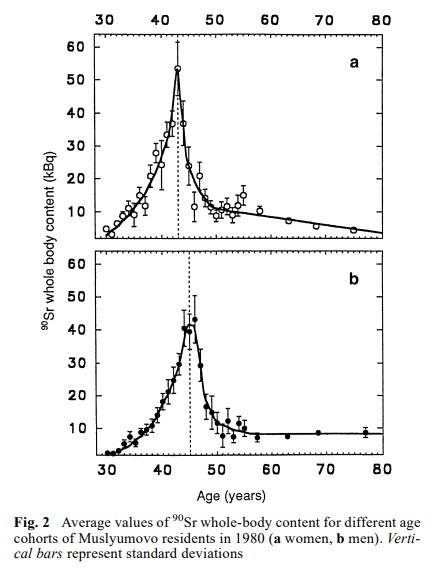


For women, strontium concentration peaked at age 43 in 1980, so they were 13 years old in 1950 when radioactive liquid releases from the Mayak reprocessing plant to the river Techa were highest.

**S3 Fig. Development of perinatal deaths and total births in Ukraine, 1981-2004.**


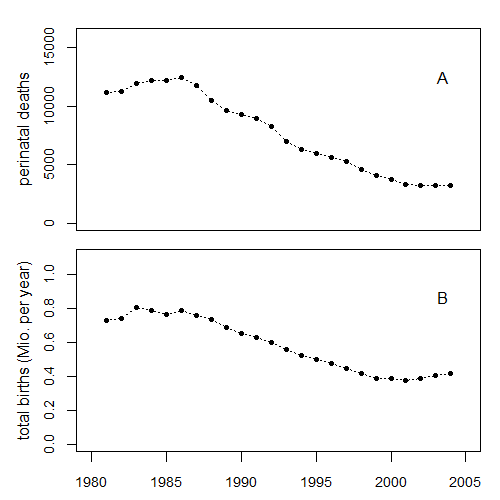


**S4 Fig. Development of perinatal deaths and total births in the oblasts of the study region**

(For the city of Kyiv, data of early neonatal deaths were only available from 1985)


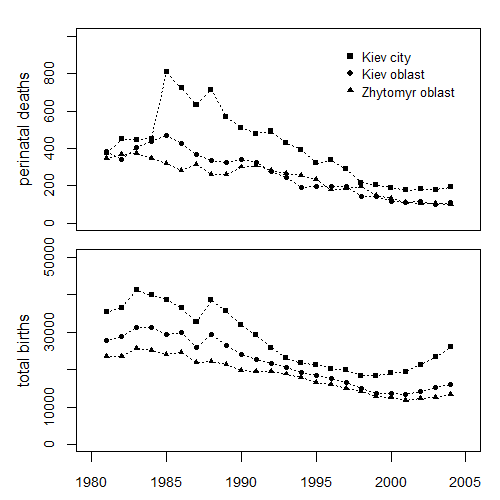


**S5 Fig. Deposited strontium-90 released in the Chernobyl accident**


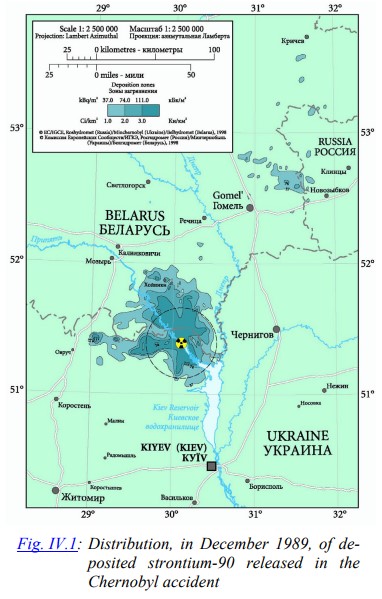


Reference:

Cort, M. De, A. R. Jones, I. M. Nazarov, Sh. D. Fridman, E. V. Kvasnikova, E. D. Stukin, and G. N. Kelly. [“The Atlas of Caesium-137 Contamination of Europe after the Chernobyl Accident.” Institute of Global Climate and Ecology, Moscow, Russia; Environment Institute, CEC Joint Research Center, Ispra, Italy; Republic Centre of Radiation and Environment Monitoring, Minsk, Belarus; Roshidromet, Moscow, Russia; CEC, DG XII.F.6, Brussels, Belgium, 1998](https://inis.iaea.org/collection/NCLCollectionStore/_Public/31/056/31056824.pdf).
