# Supplementary material for: Perinatal mortality after Chornobyl in contaminated regions of Ukraine
Source: PLoS One. 2024 May 20;19(5):e0303427. doi: 10.1371/journal.pone.0303427 (PMC11104673; doi:10.1371/journal.pone.0303427)
Supplement: S2 File — (DOCX) [file pone.0303427.s002.docx]

**Regression results**

**Ukraine**

Formula: y ~ exp(b1+b2·t+ b5/t/exp((log(t)-b6)^2/2/b7^2)+b8/t/exp((log(t)-b9)^2/2/b7^2)+b11·d87

Regression results:

| parameter | estimate | SE^1^ | t-value | p-value |
| --- | --- | --- | --- | --- |
| b1 | -3.936 | 0.009 | -454.17 | <2.0E-16 |
| b2 | -0.0377 | 0.0008 | -44.96 | 9.6E-15 |
| b5 | 1.413 | 0.112 | 12.57 | 2.9E-08 |
| b6 | 2.445 | 0.011 | 218.31 | <2.0E-16 |
| b7 | 0.121 | 0.008 | 14.68 | 5.0E-09 |
| b8 | 2.816 | 0.228 | 12.33 | 3.6E-08 |
| b9 | 2.864 | 0.011 | 251.51 | <2.0E-16 |
| b11 | 0.037 | 0.011 | 3.30 | 0.0063 |

^1^SE: Standard error; d87: dummy variable for 1987

**S1 Table. Perinatal mortality in Ukraine and fitted values**

| Year | LB | SB | NEO | Rate | Fitted values |
| --- | --- | --- | --- | --- | --- |
| 1985 | 762775 | 7841 | 4548 | 0.0161 | 0.0162 |
| 1986 | 792574 | 7873 | 4784 | 0.0158 | 0.0156 |
| 1987 | 760851 | 7504 | 4448 | 0.0156 | 0.0150 |
| 1988 | 744056 | 6710 | 4007 | 0.0143 | 0.0145 |
| 1989 | 690981 | 6143 | 3723 | 0.0142 | 0.0142 |
| 1990 | 657202 | 5724 | 3815 | 0.0144 | 0.0144 |
| 1991 | 630813 | 5338 | 3861 | 0.0145 | 0.0145 |
| 1992 | 596785 | 4818 | 3613 | 0.0140 | 0.0139 |
| 1993 | 557467 | 3990 | 3181 | 0.0128 | 0.0129 |
| 1994 | 521545 | 3707 | 2749 | 0.0123 | 0.0123 |
| 1995 | 492861 | 3409 | 2637 | 0.0122 | 0.0121 |
| 1996 | 467211 | 3218 | 2511 | 0.0122 | 0.0122 |
| 1997 | 442581 | 2966 | 2456 | 0.0122 | 0.0121 |
| 1998 | 419238 | 2597 | 2149 | 0.0113 | 0.0115 |
| 1999 | 389208 | 2353 | 1927 | 0.0109 | 0.0107 |
| 2000 | 385126 | 2076 | 1813 | 0.0100 | 0.0099 |
| 2001 | 376478 | 1830 | 1623 | 0.0091 | 0.0092 |
| 2002 | 390688 | 1837 | 1530 | 0.0086 | 0.0087 |
| 2003 | 408589 | 1969 | 1465 | 0.0084 | 0.0083 |
| 2004 | 427259 | 1986 | 1425 | 0.0079 | 0.0079 |

LB: Live births; SB: Stillbirths; NEO: Early neonatal deaths; Rate: Perinatal mortality rate

**Study region**

Formula: y ~ exp(b1+b2·t+ b5/t/exp((log(t)-b6)^2/2/b7^2)+ b8/t/exp((log(t)-b9)^2/2/b7^2)+ b11·d87+b12*d97

Regression results:

| parameter | estimate | SE | t-value | p-value |
| --- | --- | --- | --- | --- |
| b1 | -3.862 | 0.021 | -185.66 | <2E-16 |
| b2 | -0.0454 | 0.0025 | -18.09 | 1.6E-09 |
| b5 | 3.020 | 0.319 | 9.48 | 1.3E-06 |
| b6 | 2.520 | 0.020 | 123.52 | <2E-16 |
| b7 | 0.147 | 0.018 | 8.18 | 5.3E-06 |
| b8 | 4.229 | 0.581 | 7.28 | 1.6E-05 |
| b9 | 2.873 | 0.033 | 87.51 | <2E-16 |
| b11 | 0.063 | 0.025 | 2.56 | 0.0264 |
| b12 | 0.126 | 0.038 | 3.32 | 0.0068 |

SE: Standard error

**S2 Table. Perinatal mortality in the study region and fitted values**

| Year | LB | SB | NEO | Rate | Fitted values |
| --- | --- | --- | --- | --- | --- |
| 1985 | 91285 | 906 | 695 | 17.37 | 16.77 |
| 1986 | 90169 | 792 | 638 | 15.72 | 16.02 |
| 1987 | 79919 | 762 | 553 | 16.30 | 15.31 |
| 1988 | 89380 | 824 | 490 | 14.57 | 14.69 |
| 1989 | 82572 | 718 | 435 | 13.84 | 14.41 |
| 1990 | 75203 | 668 | 484 | 15.18 | 14.79 |
| 1991 | 71079 | 593 | 518 | 15.50 | 15.54 |
| 1992 | 66395 | 555 | 494 | 15.67 | 15.74 |
| 1993 | 61915 | 488 | 449 | 15.02 | 15.08 |
| 1994 | 58293 | 451 | 393 | 14.37 | 14.18 |
| 1995 | 55738 | 432 | 317 | 13.33 | 13.53 |
| 1996 | 53526 | 400 | 314 | 13.24 | 13.09 |
| 1997 | 50968 | 404 | 329 | 14.27 | 12.58 |
| 1998 | 47272 | 308 | 246 | 11.64 | 11.81 |
| 1999 | 44664 | 273 | 224 | 11.06 | 10.84 |
| 2000 | 45030 | 235 | 201 | 9.63 | 9.86 |
| 2001 | 44381 | 229 | 170 | 8.94 | 8.98 |
| 2002 | 47442 | 238 | 168 | 8.52 | 8.26 |
| 2003 | 50961 | 234 | 154 | 7.58 | 7.68 |
| 2004 | 55165 | 260 | 147 | 7.34 | 7.22 |

LB: Live births; SB: Stillbirths; NEO: Early neonatal deaths; rate: Perinatal mortality rate

**Control region**

Formula: y ~ exp(b1+b2·t+ b5/t/exp((log(t)-b6)^2/2/b7^2)+ b8/t/exp((log(t)-b9)^2/2/b7^2)+ b11·d87

Regression results:

| parameter | estimate | SE | t-value | p-value |
| --- | --- | --- | --- | --- |
| b1 | -3.946 | 0.009 | -447.41 | <2E-16 |
| b2 | -0.0368 | 0.0008 | -43.37 | <2E-16 |
| b5 | 1.243 | 0.113 | 10.96 | 1.3E-10 |
| b6 | 2.431 | 0.012 | 197.80 | <2E-16 |
| b7 | 0.117 | 0.009 | 12.52 | 9.5E-12 |
| b8 | 2.527 | 0.237 | 10.68 | 2.2E-10 |
| b9 | 2.870 | 0.012 | 235.70 | <2E-16 |
| b11 | 0.035 | 0.011 | 3.04 | 0.0058 |

SE: Standard error

**S3 Table. Data of perinatal mortality in the control region and fitted values**

| Year | LB | SB | NEO | Rate | Fitted values |
| --- | --- | --- | --- | --- | --- |
| 1985 | 671490 | 6935 | 3853 | 15.90 | 16.08 |
| 1986 | 702405 | 7081 | 4146 | 15.82 | 15.50 |
| 1987 | 680932 | 6742 | 3895 | 15.47 | 14.94 |
| 1988 | 654676 | 5886 | 3517 | 14.23 | 14.42 |
| 1989 | 608409 | 5425 | 3288 | 14.19 | 14.14 |
| 1990 | 581999 | 5056 | 3331 | 14.29 | 14.31 |
| 1991 | 559734 | 4745 | 3343 | 14.33 | 14.37 |
| 1992 | 530390 | 4263 | 3119 | 13.81 | 13.65 |
| 1993 | 495552 | 3502 | 2732 | 12.49 | 12.67 |
| 1994 | 463252 | 3256 | 2356 | 12.03 | 12.06 |
| 1995 | 437123 | 2977 | 2320 | 12.04 | 11.93 |
| 1996 | 413685 | 2818 | 2197 | 12.04 | 12.01 |
| 1997 | 391613 | 2562 | 2127 | 11.90 | 11.91 |
| 1998 | 371966 | 2289 | 1903 | 11.20 | 11.44 |
| 1999 | 344544 | 2080 | 1703 | 10.91 | 10.70 |
| 2000 | 340096 | 1841 | 1612 | 10.10 | 9.93 |
| 2001 | 332097 | 1601 | 1453 | 9.15 | 9.28 |
| 2002 | 343246 | 1599 | 1362 | 8.59 | 8.76 |
| 2003 | 357628 | 1735 | 1311 | 8.48 | 8.36 |
| 2004 | 372094 | 1726 | 1278 | 8.04 | 8.01 |

LB: Live births; SB: Stillbirths; NEO: Early neonatal deaths; rate: Perinatal mortality rate

O**dds ratios**

Model(2): y ~ exp(b1+b2·t+ b5/t/exp((log(t)-b6)^2/2/b7^2)+ b8·d97)

| parameter | estimate | SE | t-value | p-value |
| --- | --- | --- | --- | --- |
| b1 | 0.076 | 0.021 | 3.68 | 0.0025 |
| b2 | -0.0063 | 0.0018 | -3.49 | 0.0036 |
| b5 | 2.550 | 0.348 | 7.33 | 3.7E-06 |
| b6 | 2.651 | 0.034 | 78.96 | <2E-16 |
| b7 | 0.172 | 0.033 | 5.22 | 0.0001 |
| b8 | 0.130 | 0.046 | 2.82 | 0.0137 |

d97: Dummy variable for 1997

Model(3): y ~ exp(b1+b2·t+ b3·Sr+ b4·d97)

| parameter | estimate | SE | t-value | p-value |
| --- | --- | --- | --- | --- |
| b1 | 0.125 | 0.019 | 6.73 | 4.8E-06 |
| b2 | -0.0136 | 0.0019 | -7.25 | 2.0E-06 |
| b3 | 3.597 | 0.428 | 8.41 | 2.9E-07 |
| b4 | 0.101 | 0.038 | 2.68 | 0.0164 |

**S4 Table. Odds ratios and fitted values of perinatal mortality in Ukraine**

| Year | Odds1 | Odds0 | OR | Fit2 | Fit3 |
| --- | --- | --- | --- | --- | --- |
| 1985 | 0.01767 | 0.01616 | 1.094 | 1.045 | 1.058 |
| 1986 | 0.01597 | 0.01608 | 0.993 | 1.039 | 1.044 |
| 1987 | 0.01657 | 0.01571 | 1.055 | 1.032 | 1.031 |
| 1988 | 0.01478 | 0.01444 | 1.024 | 1.027 | 1.021 |
| 1989 | 0.01404 | 0.01440 | 0.975 | 1.028 | 1.020 |
| 1990 | 0.01542 | 0.01449 | 1.064 | 1.047 | 1.038 |
| 1991 | 0.01575 | 0.01454 | 1.083 | 1.089 | 1.078 |
| 1992 | 0.01592 | 0.01400 | 1.137 | 1.143 | 1.129 |
| 1993 | 0.01524 | 0.01265 | 1.205 | 1.182 | 1.167 |
| 1994 | 0.01458 | 0.01218 | 1.197 | 1.184 | 1.177 |
| 1995 | 0.01351 | 0.01218 | 1.109 | 1.152 | 1.158 |
| 1996 | 0.01342 | 0.01219 | 1.101 | 1.104 | 1.123 |
| 1997 | 0.01448 | 0.01204 | 1.202 | 1.056 | 1.087 |
| 1998 | 0.01178 | 0.01133 | 1.040 | 1.016 | 1.054 |
| 1999 | 0.01118 | 0.01103 | 1.014 | 0.987 | 1.023 |
| 2000 | 0.00973 | 0.01020 | 0.953 | 0.967 | 0.994 |
| 2001 | 0.00902 | 0.00924 | 0.977 | 0.953 | 0.965 |
| 2002 | 0.00859 | 0.00866 | 0.992 | 0.943 | 0.937 |
| 2003 | 0.00764 | 0.00855 | 0.893 | 0.935 | 0.912 |
| 2004 | 0.00740 | 0.00810 | 0.913 | 0.928 | 0.888 |

Odds1, Odds0: Odds of perinatal mortality in study and control region; OR: odds ratio; :
Fit2, Fit3: Fitted values with models (2) and (3), respectively.
