# Supplementary material for: Perinatal mortality after Chornobyl in contaminated regions of Ukraine
Source: PLoS One. 2024 May 20;19(5):e0303427. doi: 10.1371/journal.pone.0303427 (PMC11104673; doi:10.1371/journal.pone.0303427)
Supplement: S1 Dataset — (DOCX) [file pone.0303427.s003.docx]

**Perinatal mortality data**

**S1 Table. Perinatal mortality from Ukraine and the oblasts of the study region**

|  | Zhytomyr Oblast | | | Kyiv oblast | | |
| --- | --- | --- | --- | --- | --- | --- |
| year | LB | SB | NEO | LB | SB | NEO |
| 1981 | 23231 | 255 | 93 | 27621 | 254 | 128 |
| 1982 | 23237 | 257 | 109 | 28649 | 219 | 124 |
| 1983 | 25401 | 264 | 109 | 30953 | 283 | 121 |
| 1984 | 24788 | 258 | 91 | 31006 | 286 | 152 |
| 1985 | 23909 | 214 | 107 | 29086 | 293 | 179 |
| 1986 | 24489 | 190 | 93 | 29559 | 275 | 150 |
| 1987 | 21763 | 184 | 129 | 25798 | 251 | 117 |
| 1988 | 21992 | 170 | 93 | 29249 | 227 | 111 |
| 1989 | 21115 | 165 | 96 | 26173 | 208 | 116 |
| 1990 | 19634 | 185 | 116 | 23937 | 202 | 137 |
| 1991 | 19477 | 165 | 145 | 22653 | 178 | 145 |
| 1992 | 19238 | 171 | 111 | 21538 | 160 | 116 |
| 1993 | 18690 | 159 | 106 | 20393 | 126 | 118 |
| 1994 | 17695 | 143 | 114 | 19113 | 126 | 68 |
| 1995 | 16344 | 136 | 97 | 18300 | 125 | 70 |
| 1996 | 15925 | 117 | 61 | 17562 | 118 | 81 |
| 1997 | 14866 | 113 | 71 | 16470 | 130 | 66 |
| 1998 | 14118 | 117 | 78 | 14873 | 79 | 62 |
| 1999 | 12669 | 97 | 51 | 13672 | 77 | 69 |
| 2000 | 12589 | 79 | 52 | 13487 | 62 | 54 |
| 2001 | 11733 | 62 | 51 | 13288 | 56 | 53 |
| 2002 | 12292 | 65 | 42 | 13994 | 61 | 56 |
| 2003 | 12607 | 60 | 48 | 15079 | 67 | 34 |
| 2004 | 13265 | 62 | 40 | 16016 | 72 | 39 |
| 2005 | 12904 | 83 | 70 | 16460 | 75 | 52 |
| 2006 | 13654 | 67 | 69 | 17383 | 83 | 96 |

Year: calendar year; LB: live births, SB: stillbirths, NEO: early neonatal deaths

**S1 Table continued**

|  | Kyiv City | | | Ukraine | | |
| --- | --- | --- | --- | --- | --- | --- |
| year | LB | SB | NEO | LB | SB | NEO |
| 1981 | 34995 | 375 | 0 | 733183 | 7220 | 3959 |
| 1982 | 36107 | 450 | 0 | 745591 | 7432 | 3819 |
| 1983 | 40803 | 446 | 0 | 807111 | 7722 | 4329 |
| 1984 | 39451 | 451 | 0 | 792035 | 7840 | 4448 |
| 1985 | 38290 | 399 | 409 | 762775 | 7841 | 4548 |
| 1986 | 36121 | 327 | 395 | 792574 | 7873 | 4784 |
| 1987 | 32358 | 327 | 307 | 760851 | 7504 | 4448 |
| 1988 | 38139 | 427 | 286 | 744056 | 6710 | 4007 |
| 1989 | 35284 | 345 | 223 | 690981 | 6143 | 3723 |
| 1990 | 31632 | 281 | 231 | 657202 | 5724 | 3815 |
| 1991 | 28949 | 250 | 228 | 630813 | 5338 | 3861 |
| 1992 | 25619 | 224 | 267 | 596785 | 4818 | 3613 |
| 1993 | 22832 | 203 | 225 | 557467 | 3990 | 3181 |
| 1994 | 21485 | 182 | 211 | 521545 | 3707 | 2749 |
| 1995 | 21094 | 171 | 150 | 492861 | 3409 | 2637 |
| 1996 | 20039 | 165 | 172 | 467211 | 3218 | 2511 |
| 1997 | 19632 | 161 | 192 | 442581 | 2966 | 2456 |
| 1998 | 18281 | 112 | 106 | 419238 | 2597 | 2149 |
| 1999 | 18323 | 99 | 104 | 389208 | 2353 | 1927 |
| 2000 | 18954 | 94 | 95 | 385126 | 2076 | 1813 |
| 2001 | 19360 | 111 | 66 | 376478 | 1830 | 1623 |
| 2002 | 21156 | 112 | 70 | 390688 | 1837 | 1530 |
| 2003 | 23275 | 107 | 72 | 408589 | 1969 | 1465 |
| 2004 | 25884 | 126 | 68 | 427259 | 1986 | 1425 |
| 2005 | 26258 | 115 | 127 | 426086 | 2242 | 2431 |
| 2006 | 28101 | 112 | 151 | 460427 | 2714 | 2566 |

**S2 Table. Perinatal mortality data from oblast Poltava (Ukraine)**

| Year | LB | SB | NEO |
| --- | --- | --- | --- |
| 1981 | 21866 | 221 | 127 |
| 1982 | 22793 | 225 | 110 |
| 1983 | **23983** | 266 | 113 |
| 1984 | 23889 | 278 | 112 |
| 1985 | 22856 | 230 | 124 |
| 1986 | 24550 | 260 | 139 |
| 1987 | 24111 | 252 | 118 |
| 1988 | 23491 | 200 | 106 |
| 1989 | 21681 | 171 | 95 |
| 1990 | 20798 | 202 | 109 |
| 1991 | 20279 | 169 | 79 |
| 1992 | 19042 | 159 | 93 |
| 1993 | 17651 | 123 | 73 |
| 1994 | 16665 | 114 | 62 |
| 1995 | 15835 | 106 | 68 |
| 1996 | 15164 | 103 | 66 |
| 1997 | 14021 | 95 | 77 |
| 1998 | 13176 | 84 | 72 |
| 1999 | 12228 | 67 | 53 |
| 2000 | 11641 | 53 | 34 |
| 2001 | 11133 | 51 | 36 |
| 2002 | 11546 | 48 | 28 |
| 2003 | 11835 | 47 | 28 |
| 2004 | 11792 | 57 | 26 |

Year: calendar year; LB: live births, SB: stillbirths, NEO: early neonatal deaths

Data provided by N. Omelyanets, Kyiv

**S3 Table. Perinatal mortality data from oblast Rivne (Ukraine)**

| Year | LB | SB | NEO |
| --- | --- | --- | --- |
| 1980 | 20286 | 214 | 82 |
| 1981 | 20394 | 203 | 102 |
| 1982 | 20567 | 200 | 93 |
| 1983 | 21607 | 179 | 102 |
| 1984 | 21246 | 203 | 89 |
| 1985 | 20788 | 201 | 105 |
| 1986 | 22104 | 219 | 153 |
| 1987 | 21112 | 210 | 122 |
| 1988 | 20584 | 205 | 71 |
| 1989 | 19270 | 151 | 81 |
| 1990 | 18519 | 175 | 101 |
| 1991 | 17954 | 171 | 82 |
| 1992 | 18283 | 161 | 77 |
| 1993 | 18212 | 138 | 94 |
| 1994 | 17289 | 135 | 81 |
| 1995 | 16469 | 116 | 82 |
| 1996 | 15782 | 100 | 54 |
| 1997 | 15770 | 110 | 54 |
| 1998 | 15071 | 86 | 65 |
| 1999 | 14188 | 91 | 61 |
| 2000 | 13898 | 85 | 77 |
| 2001 | 13252 | 58 | 41 |
| 2002 | 13407 | 69 | 50 |
| 2003 | 13940 | 80 | 36 |
| 2004 | 14558 | 54 | 42 |
| 2005 | 14483 | 65 | 67 |
| 2006 | 15758 | 59 | 35 |
| 2007 | 15759 | 89 | 70 |
| 2008 | 17089 | 99 | 55 |
| 2009 | 17544 | 99 | 63 |
| 2010 | 17074 | 117 | 57 |
| 2011 | 17697 | 114 | 71 |
| 2012 | 18316 | 116 | 67 |

Year: calendar year; LB: live births, SB: stillbirths, NEO: early neonatal deaths

Data provided by Omni-Net Ukraine, http://ukraineomni.org/en/about_eng/

**S4 Table. Perinatal mortality data from Belarus, the oblast Gomel, and Minsk city**

|  | Belarus | | | Gomel Oblast | | | Minsk City | | |
| --- | --- | --- | --- | --- | --- | --- | --- | --- | --- |
| Year | LB | SB | NEO | LB | SB | NEO | LB | SB | NEO |
| 1985 | 165024 | 1267 | 948 | 28806 | 219 | 156 | 27436 | 225 | 222 |
| 1986 | 171674 | 1271 | 1043 | 28849 | 208 | 168 | 28258 | 253 | 218 |
| 1987 | 162890 | 1247 | 959 | 24813 | 187 | 152 | 27273 | 249 | 223 |
| 1988 | 163247 | 1156 | 950 | 27550 | 200 | 138 | 27364 | 262 | 193 |
| 1989 | 153435 | 1011 | 830 | 25868 | 171 | 136 | 25638 | 228 | 175 |
| 1990 | 142109 | 954 | 830 | 22301 | 161 | 151 | 23508 | 203 | 174 |
| 1991 | 132097 | 876 | 725 | 20433 | 151 | 138 | 20919 | 181 | 145 |
| 1992 | 127920 | 753 | 701 | 20294 | 138 | 108 | 19430 | 138 | 134 |
| 1993 | 117361 | 595 | 594 | 19244 | 119 | 106 | 17299 | 102 | 127 |
| 1994 | 110503 | 715 | 673 | 17940 | 121 | 138 | 16267 | 150 | 124 |
| 1995 | 101155 | 645 | 599 | 16146 | 123 | 109 | 15125 | 137 | 111 |
| 1996 | 95831 | 521 | 509 | 15136 | 81 | 101 | 14580 | 102 | 94 |
| 1997 | 89561 | 526 | 401 | 14398 | 103 | 70 | 13199 | 79 | 32 |

Year: calendar year; LB: live births, SB: stillbirths, NEO: early neonatal deaths

**S5 Table. Numbers of live births by maternal age in Saint Petersburg, 1990-1999**

| Age (years) | 1990-1994 | 1995-1999 |
| --- | --- | --- |
| 15 | 358 | 256 |
| 16 | 1037 | 850 |
| 17 | 3105 | 2132 |
| 18 | 6678 | 4482 |
| 19 | 11403 | 7516 |
| 20 | 15426 | 10629 |
| 21 | 16486 | 11394 |
| 22 | 16178 | 12075 |
| 23 | 15295 | 11566 |
| 24 | 13849 | 12029 |
| 25 | 12430 | 11973 |
| 26 | 12132 | 10128 |
| 27 | 10522 | 9282 |
| 28 | 9944 | 8027 |
| 29 | 10093 | 6778 |
| 30 | 9408 | 6385 |
| 31 | 8105 | 5451 |
| 32 | 6821 | 5030 |
| 33 | 5500 | 4326 |
| 34 | 4644 | 3769 |
| 35 | 3950 | 3395 |
| 36 | 3488 | 2815 |
| 37 | 2531 | 2007 |
| 38 | 1868 | 1567 |
| 39 | 1366 | 1265 |
| 40 | 1035 | 847 |
| 41 | 790 | 561 |
| 42 | 429 | 364 |
| 43 | 259 | 191 |
| 44 | 54 | 60 |

Data obtained from Dr. Natalia Kovaleva, Saint Petersburg University
