# Supplementary material for: Perinatal mortality after Chornobyl in contaminated regions of Ukraine
Source: PLoS One. 2024 May 20;19(5):e0303427. doi: 10.1371/journal.pone.0303427 (PMC11104673; doi:10.1371/journal.pone.0303427)
Supplement: Attachment — Submitted filename: Response to Reviewers.docx [file pone.0303427.s004.docx]

**Response to Reviewers**

(highlighted in blue)

**Reviewer #1:**
The authors have excellently inferred based on theoretical modelling that the observed increase in perinatal mortality in the 1990s may be a late effect of incorporated radioactive strontium-90 on the immune system of pregnant women. Though the results are not fully conclusive and further studies may be warranted in future, the manuscript will lay the foundation for such research and is hence acceptable for publication as it is. The reviewer congratulates the author for this thought-provoking study.

**Reviewer #2:**
It is self-evident that the author is a competent investigator and has produced a thorough report. However, from a biologic point of view, statistical analyses cannot improve data flaws nor interpretation flaws. We believe that the report focuses attention on an important issue - death as an end point of radiation "sickness" "syndrome". Incorporated radionuclides impact physiology and homeostasis and may enhance lethal effects of extemporaneous causes rendering infants more label, ultimately reflected in mortality. Incorporation of radionuclides is gradual by women, pregnant women, and by their infants. Also, noteworthy is that at the time of the reported peak of mortality, in Ukraine, the definitions of stillbirth-livebirth were modified. In summary, we believe this report is important and that the author or authors can easily restructure the presentation. In this spirit we offer the following suggestions.

AK: The definition of stillbirths, using birthweight as a criterion, seems to have changed after 2004 in Ukraine, not around 1993 when there is a peak in odds ratios of perinatal mortality.

1. The author indicates that the analysis of the data provided by Dr. N. Omelyanets was to be published jointly. The author submits this report without Dr. N. Omelyanets and instead acknowledges his contribution. A comment clarifying this discrepancy would be welcome.

AK: Prof. Omelyanets died in 2021.

2. The control data includes Rivne oblast which in turn includes Rivne Polissia. This region is considered by multiple experts to be among the most polluted by Chornobyl radionuclides. Furthermore, the soils in Rivne Polissia have the highest transfer index of radionuclides from soil to biota (see reports by Likhtarev et al.). This fact enhances the biologic impacts of incorporated radionuclides in this area. Rivne Polissia should not be included as a component of a "control" population.

AK: When I asked for the data in 2006, I was not aware of the high contamination of oblast Rivne. As the study region I defined the two oblasts Zhytomyr and oblast Kyiv, home of the Chornobyl NPP, the adjacent oblast Zhytomyr, and the city of Kyiv which is in the middle of the oblast Kyiv. But the oblast Rivne is quite distant from the Chornobyl NPP. My study is on possible health effects from strontium, not cesium. Strontium in the fallout is less volatile than cesium and was deposited in a much smaller area than cesium. A map of the strontium surface contamination is provided in my supporting information file.

3. The incorporation of radionuclides represents admixtures. Generally, the proportion of incorporated Cs-137 is greater than of Sr-90. The reasons why the author is entirely focused on one radionuclide is at best questionable. Perhaps, there are good reasons for this emphasis and if so, they should be stated more clearly.

AK: In my study I try to find a mechanism to explain an increase in perinatal mortality in the 1990s. The contamination of food with cesium-137 was highest in 1986 and decreased slowly in the following years. My analysis finds a significant 3.8% increase (p=0.006) in perinatal mortality in Ukraine in 1987, but no increase in 1988 or 1989. However, a sharp rise is observed in 1990 with a first maximum around 1993. I don’t see how this increase can be explained by cesium-90, the only other isotope with long enough half-life.

From: Cort, M. De, A. R. Jones, I. M. Nazarov, Sh. D. Fridman, E. V. Kvasnikova, E. D. Stukin, and G. N. Kelly. [“The Atlas of Caesium-137 Contamination of Europe after the Chernobyl Accident.” Institute of Global Climate and Ecology, Moscow, Russia; Environment Institute, CEC Joint Research Center, Ispra, Italy; Republic Centre of Radiation and Environment Monitoring, Minsk, Belarus; Roshidromet, Moscow, Russia; CEC, DG XII.F.6, Brussels, Belgium, 1998](https://inis.iaea.org/collection/NCLCollectionStore/_Public/31/056/31056824.pdf).

AK: To investigate whether adding the oblast Rivne to the study region (and removing it from the control region) influences the result, I determined the odds ratios, i.e., the ratio of perinatal mortality rates in the oblast Rivne to the rates in my control region excluding the oblast Rivne. The figure below shows trend of the odds ratios in 1981-2004. No effect of the bell-shaped excess term or the strontium term was found.

Trend of perinatal mortality rates in the oblast Rivne, and in the modified control region. The lower panel shows the ratio of the perinatal mortality rates in the oblast Rivne to the rates in the control region (Ukraine minus, the oblasts Zhytomyr, Kyiv, Rivne, and the city of Kyiv)

4. The report concerns mortality and, as is stands, it almost conveys the notion of a statistical exercise which belongs in a supplement.

AK: Here I rely on the advice of the editor(s).

Best regards

Alfred Körblein
